# Immune Checkpoint Inhibitors Plus Single-Agent Chemotherapy for Advanced Non-Small-Cell Lung Cancer After Resistance to EGFR-TKI

**DOI:** 10.3389/fonc.2021.700023

**Published:** 2021-09-20

**Authors:** Haiyi Deng, Xinqing Lin, Xiaohong Xie, Yilin Yang, Liqiang Wang, Jianhui Wu, Ming Liu, Zhanhong Xie, Yinyin Qin, Chengzhi Zhou

**Affiliations:** State Key Laboratory of Respiratory Disease, National Clinical Research Centre for Respiratory Disease, Guangzhou Institute of Respiratory Health, First Affiliated Hospital, Guangzhou Medical University, Guangzhou, China

**Keywords:** epidermal growth factor receptor gene (EGFR), immune checkpoint inhibitor, immunotherapy, non-small-cell lung cancer, single-agent chemotherapy

## Abstract

**Purpose:**

Platinum-based chemotherapy remains the classic treatment option for patients with advanced non-small-cell lung cancer (NSCLC) who progress while receiving treatment with epidermal growth factor receptor-tyrosine kinase inhibitors (EGFR-TKIs). In this study, we analyzed real-world outcomes of treatment with immune checkpoint inhibitors (ICIs) combined with platinum-free chemotherapy in patients with NSCLC after developing resistance to EGFR-TKIs.

**Methods:**

This retrospective study included patients with mutation-positive NSCLC after developing resistance to EGFR-TKIs. Patients who received chemotherapy alone plus ICIs with or without anti-angiogenic drugs (cohort A) or platinum-based chemotherapy (cohort B) between February 2019 and August 2020 were enrolled. Clinical characteristics, EGFR mutation status, response to therapy, and adverse events (AEs) were retrospectively analyzed.

**Results:**

Seventeen patients were eligible and included in the analysis, including 8 in cohort A and 9 in cohort B. After a median follow-up of 7.6 months, the median progression-free survival was 6.5 months [95% confidence interval (CI), 6.1 to 7.0] in cohort A and 3.6 months (95% CI, 1.3–5.8) in cohort B (hazard ratios, 0.22; 95% CI, 0.05–0.93; P = 0.039). The overall response and disease control rates were 50% and 100% in cohort A, and 22% and 89% in cohort B, respectively. Adverse events of grade 3 or higher occurred in 25% of the patients in cohort A and in 33.3% of the patients in cohort B.

**Conclusion:**

ICIs plus platinum-free, single-agent chemotherapy provides promising progression-free survival and overall response rate benefit, along with a low rate of severe AEs in patients with EGFR-TKI-resistant advanced NSCLC.

## Introduction

Lung cancer is the first-leading cause of cancer-related death worldwide (1). The vast majority (85%) of lung cancer cases are non-small-cell lung cancer (NSCLC), and approximately 50% of them harbor epidermal growth factor receptor gene (EGFR) mutations in Asia (2). Multiple clinical studies have confirmed the significant response of patients with EGFR mutation to EGFR-tyrosine kinase inhibitors (EGFR-TKIs) (3). According to the current guidelines, the use of EGFR-TKIs has been recommended for the first-line treatment of advanced NSCLC with EGFR mutation. However, these patients develop resistance to EGFR-TKIs after 9–14 months. Approximately 50% of the patients with resistance to first- and second-generation TKIs have T790M mutations, which can be treated with third-generation TKIs (4). Unfortunately, these patients also develop resistance to third-generation EGFR-TKIs after approximately 1 year. According to the guidelines, chemotherapy is generally selected for follow-up treatment of T790M-negative or T790M-positive patients with resistance to third-generation TKIs; however, the efficacy of this regimen is unsatisfactory.

Immune checkpoint inhibitors (ICIs) offer a survival benefit to NSCLC patients without EGFR mutation, but not to those with EGFR mutations (5). The potential benefits of ICIs to patients with resistance to EGFR-TKIs are currently being explored. A meta-analysis showed that ICI alone does not exert a better effect than chemotherapy for patients who have progressed after treatment with TKIs (6). A phase II study (CT18, NCT03513666) of toripalimab combined with pemetrexed and carboplatin showed favorable efficacy (7). IMpower 150, which enrolled patients with EGFR mutation, demonstrated that ICI combined with chemotherapy and an anti-angiogenic drug prolonged progression-free survival (PFS) *versus* chemotherapy plus an anti-angiogenic drug (8). However, these two clinical studies also showed higher proportion of grades 3–5 adverse events (AEs).

A study showed that the immunomodulatory effects of pemetrexed or paclitaxel appeared to be reduced when combined with platinum (9). In addition, CheckMate 9LA confirmed that dual immunotherapy combined with limited chemotherapy exerts a good effect (10). The addition of chemotherapy to immunotherapy should not be restricted to the regimen of four courses of platinum-based chemotherapy. Our previous study proposed the concept of “chemo-reform”: the addition of post-reform chemotherapy to immunotherapy, including single-drug chemotherapy (without platinum), platinum alone, low-dose chemotherapy, chemotherapy with adjusted course, or cycle interval–adjusted chemotherapy (11). This retrospective study was designed to assess the efficacy and safety of ICIs combined with single-drug chemotherapy without platinum in patients with EGFR-TKI-resistant advanced NSCLC in a real-world setting.

## Patients and Methods

### Patients

Eligible patients with incurable NSCLC were treated with ICIs combined with single-drug chemotherapy without platinum, with or without anti-angiogenic treatment (cohort A) or platinum-containing chemotherapy (cohort B) at the First Affiliated Hospital of Guangzhou Medical University (Guangzhou, China) between February 2019 and August 2020. All patients had incurable advanced or metastatic NSCLC [unresectable stage III or IV according to the 8th edition TNM classification (12)]. Patients had to have sensitizing EGFR mutations [exon 19 deletions (Del19); exon 21 L858R mutation (L858R)] detected at the initial biopsy, clinical or radiological progression after at least one treatment with EGFR-TKIs, and no mutations that can be targeted for treatment. There was no upper limit regarding the number of prior treatments with EGFR-TKIs or systemic therapies. This study was approved by the local Ethics Committee of the First Affiliated Hospital of Guangzhou Medical University.

### Data Collection and Outcome Assessment

The following information was retrospectively collected from the medical records of the patients: patient demographics, prior treatments with EGFR-TKIs or systemic therapies, lines of immunotherapy, Eastern Cooperative Oncology Group (ECOG) performance status (PS), EGFR mutation type, programmed death-ligand 1 (PD-L1) tumor proportion score (TPS), tumor imaging, tumor response to therapy, and AEs. The ECOG PS was evaluated prior to treatment. PD-L1 expression was tested by anti-human PD-L1 (Dako 22C3) according to the manufacturer’s recommendations, using 4–5 μm formalin-fixed and paraffin-embedded (FFPE) sections. The cutoff value was 1% for PD-L1 positivity or negativity (PD-L1+/−). Tumor response was assessed in accordance with the Response Evaluation Criteria in Solid Tumors (RECIST version 1.1) (13). The objective response rate (ORR) was defined as the percentage of patients who exhibited response (complete or partial). The disease control rate (DCR) corresponds to all cases with complete response (CR), partial response (PR), and stable disease (SD). PFS was defined as the time from therapy initiation to disease progression or death. AEs were graded according to the National Cancer Institute Common Terminology Criteria for Adverse Events, version 4.0.

### Statistical Analysis

Continuous and categorical data were summarized as medians (ranges) and frequencies (percentages), respectively. An independent-samples t-test or the Mann-Whitney U test was used to analyze continuous variables. Differences in categorical variables were assessed using either Chi-square (χ2) or Fisher’s exact test. We used the binomial exact method to evaluate the ORR and DCR with 95% confidence intervals (CIs). The Kaplan–Meier method was used to evaluate PFS with 95% CI. Statistical tests were two-sided, and a P-value <0.05 denoted statistically significant difference. Statistical analyses were performed using the IBM SPSS Statistics version 25.0 (Armonk, NY, USA) software.

## Results

### Patients

In total, 17 patients were eligible and enrolled in the study. Patients’ demographics are summarized in **Tables 1** and **2**. The median age of all patients was 59 (range: 42–73)years; nine patients (52.9%) were males and 82.4% had never smoked. All patients were adenocarcinoma. EGFR mutation status in the initial biopsy was Del19 and L858R in 11 and 6 patients, respectively. Nine patients were positive for the T790M mutation. Third or later lines were reported in 76.5% of patients, and 11 patients were treated with at least two EGFR-TKIs. For the 14 participants with available PD-L1 TPS values, 6 (42.8%), 4 (28.6%), and 4 (28.6%) patients had TPS values less than 1%, 1–49%, and ≥50%.

Cohort A included 8 (47.1%) patients and cohort B had 9 (52.9%) patients. In the cohort A, 2 patients received ICI combined with single agent chemotherapy and 6 patients received ICI combined with single-agent chemotherapy and anti-angiogenesis therapy. Baseline demographic and disease characteristics were no significant difference between groups (**Table 1**).

**Table 1:** Characteristics of patients at baseline.

Characteristics	Cohort A (N = 8)	Cohort B (N = 9)	P value
Age,y; median (Range)	66 (53-73)	58 (42-71)	0.18
Sex (male/ female)	4/4	5/4	1
Smoking status			0.58
Current/former	2 (25)	1 (11.1)	
Never	6 (75)	8 (88.9)	
ECOG PS (at initiation of ICI/chemotherapy)			0.58
0	2 (25)	1 (11.1)	
1	6 (75)	8 (88.9)	
Disease status			0.47
III B	1 (12.5)	0	
IV	7 (87.5)	9 (100)	
Site of metastasis (at initiation of ICI/chemotherapy)			
Brain	3 (37.5)	4 (44.4)	1
Liver	3 (37.5)	2 (22.2)	0.62
EGFR mutation status (initial biopsy/pre-TKI)			1
Del19	5 (62.5)	6 (66.7)	
L858R	3 (37.5)	3 (33.3)	
T790M status (rebiopsy/post-TKI)			0.64
Positive	5 (62.5)	4 (44.4)	
Negative	3 (37.5)	5 (55.6)	
Prior treatment lines			0.57
1	2 (25)	2 (22.2)	
2	3 (37.5)	6 (66.7)	
3	3 (37.5)	1 (11.1)	
PD-L1 TPS			0.11
<1%	3 (37.5)	3 (33.3)	
1-49%	4 (50)	0	
≥50%	1 (12.5)	3 (33.3)	
Unknown	0	3 (33.3)	

ECOG PS, Eastern Cooperative Oncology Group performance status; EGFR, epidermal growth factor receptor gene; ICI, immune checkpoint inhibitor; TKI, tyrosine kinase inhibitor; Del19, exon 19 deletions; PD-L1 TPS, programmed death-ligand 1 tumor proportion score.

### Efficacy

**Figure 1:**
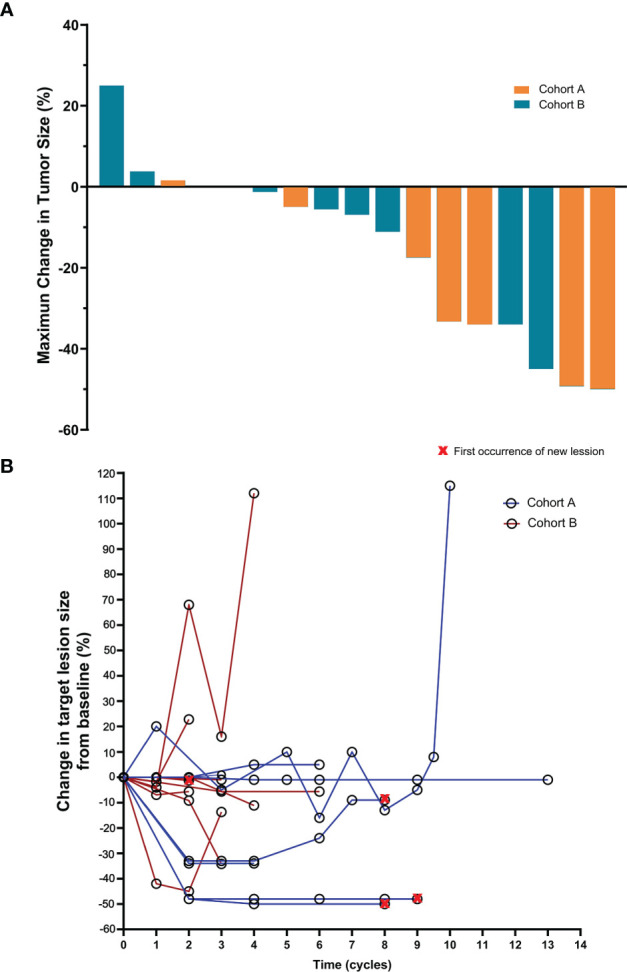
Maximum percent change from baseline in the sum of the diameter of the longest target lesion according to the Response Evaluation Criteria in Solid Tumors (RECIST) version 1.1 in patients with measurable disease at baseline **(A)**. Positive and negative change in tumor size indicates tumor growth and reduction, respectively. Percent change in target lesion tumor burden from baseline and throughout the course of immunotherapy **(B)**.

**Figure 2:**
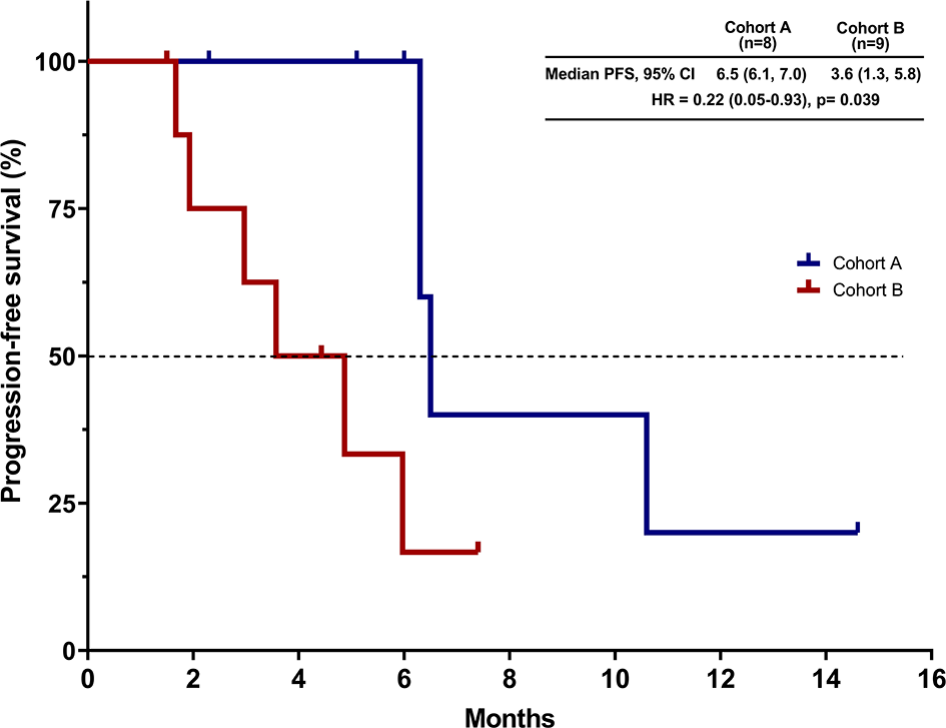
Kaplan–Meier curve for progression-free survival (PFS). CI, confidence interval; HR, hazard ratio.

A total of 17 patients were evaluable for response. Partial responses were observed in 4 of 8 patients in cohort A and in 2 of 9 patients in cohort B. None of the patients had CR as their best reponse. The confirmed ORR was 50% (95% CI, 16–84) in cohort A and 22% (95% CI, 3–60) in cohort B (P = 0.34; **Table 2** and **Figure 1**). The DCR was 100% in cohort A and 88.9% in cohort B. The overall median follow-up for this analysis was 7.6 months. With 10 events of progression or death, the median PFS was 6.5 months (95% CI, 6.1 to 7.0) in cohort A and 3.6 months (95% CI, 1.3–5.8) in cohort B [hazard ratios (HR) for PFS, 0.22; 95% CI, 0.05–0.93; P = 0.039; **Figure 2**]. The 6-month PFS rate was 60.0% in cohort A and 16.7% in cohort B. During the follow-up, the median overall survival was not reached.

**Table 2:** Characteristics of patients with EGFR-TKIs resistance who received immunotherapy or chemotherapy.

Patients	Gender	Age	Smoking status	EGFR mutation	T790M status (post-TKI)	Prior treatment lines	PFS on first TKI(months)	PD-L1 Expression level (%)	Response to ICI or chemotherapy
A-1	Female	54	Never	Del19	Negative	3	6.0	50	SD
A-2	Female	58	Never	L858R	Negative	1	10.3	2	SD
A-3	Female	68	Never	L858R	Positive	2	6.7	2	SD
A-4	Male	53	Former	Del19	Positive	3	9.7	<1	PR
A-5	Male	70	Former	L858R	Negative	1	4.6	<1	PR
A-6	Male	66	Never	Del19	Positive	2	11.9	10	PR
A-7	Male	66	Never	Del19	Positive	3	10.3	<1	SD
A-8	Female	73	Never	Del19	Positive	2	7.4	5	PR
B-1	Female	70	Never	L858R	Negative	2	3.7	<1	SD
B-2	Female	55	Never	Del19	Positive	2	8.1	<1	SD
B-3	Female	42	Never	Del19	Negative	1	4.6	80	PR
B-4	Male	59	Never	L858R	Negative	1	4.9	80	PR
B-5	Female	59	Never	Del19	Negative	2	6.1	<1	PD
B-6	Male	57	Never	Del19	Negative	2	9.5	NA	SD
B-7	Male	58	Never	Del19	Positive	2	10.9	NA	SD
B-8	Male	71	Former	L858R	Positive	2	12.2	NA	SD
B-9	Male	48	Never	Del19	Positive	3	7.6	50	SD

EGFR, epidermal growth factor receptor gene; Del19, exon 19 deletions; TKI, tyrosine kinase inhibitor; PFS, progression-free survival; PD-L1 , programmed death-ligand 1; ICI, immune checkpoint inhibitor, A-, cohort A; B-, cohort B; NA, unknown.

### Safety

A summary of the safety data of all treated patients is shown in **Table 3**. During treatment, AEs of any grade, regardless of attribution to treatment by the investigator, occurred in all patients. AEs of grade 3 or higher occurred in 25% of the patients in cohort A and in 33.3% of the patients in cohort B. There was no occurrence of toxicity-related deaths. None of the AEs led to discontinuation of treatment with ICIs in cohort A. The most common AEs in cohort A were anemia (75%), constipation (75%), and fatigue (50%), and the most common AEs in cohort B were anemia (77.8%) and decreased neutrophil count (33.3%).

**Table 3:** Adverse Events of Any Cause.

Event	Cohort A (N = 8)		Cohort B (N = 9)	
	Any Grade	Grade 3 or 4	Any Grade	Grade 3 or 4
Any event	8 (100)	2 (25)	9 (100)	3 (33.3)
Anemia	6 (75)	2 (25)	7 (77.8)	1 (11.1)
Constipation	6 (75)	0	0	0
Fatigue	4 (50)	0	1 (11.1)	0
Decreased appetite	3 (37.5)	0	2 (22.2)	0
Skin reactions	3 (37.5)	0	2 (22.2)	0
Peripheral edema	3 (37.5)	0	1 (11.1)	0
Mucosal inflammation	3 (37.5)	0	1 (11.1)	0
Hyperthyroidism	2 (25)	0	1 (11.1)	0
Diabetes	2 (25)	0	0	0
Hepatitis	2 (25)	0	2 (22.2)	1 (11.1)
Dizziness	2 (25)	0	0	0
Neutrophil count decreased	1 (12.5)	0	3 (33.3)	1 (11.1)
Pneumonitis	1 (12.5)	0	0	0
Vomiting	1 (12.5)	0	0	0
Hypothyroidism	1 (12.5)	0	0	0
Nephritis	1 (12.5)	0	0	0

## Discussion

The results of this study involving patients with advanced NSCLC patients after TKI acquired resistance showed that ICIs plus platinum-free chemotherapy, as compared with platinum-containing chemotherapy, prolonged median progression-free survival by 2.9 months (6.5 *vs.* 3.6 months).The risk of disease progression or death was 22% lower in cohort A than in cohort B. The ORR was higher in the in cohort A than in cohort B (50% *vs.* 22%). To our knowledge, this is the first study to demonstrate the efficacy and safety of immunotherapy combined with platinum-free chemotherapy in such patients.

The subsequent therapy of EGFR positive patients after TKI resistance is a conundrum. *In vitro*, PD-1 inhibitor prolonged the survival of mice with EGFR-mutant lung cancer by enhancing effector T cell function and reducing the level of tumor-promoting cytokines (14). However, previous studies showed ICI monotherapy has no survival benefit than docetaxel in the treatment of TKI resistant NSCLC patients (6), which may be associated with low rates of concurrent PD-L1 expression and CD8(+) TILs within the tumor microenvironment (15). Chemotherapy and immunotherapy had synergistic effects by upregulating PD-L1 expression (16).

Platinum-based chemotherapy is regarded as the standard treatment for EGFR-TKI resistant patients and is an appropriate comparator for this study. Although there were confounding effects compared with historical data, the outcomes of cohort B were consistent with the expectations based on previous studies (17, 18). Also, the outcomes of cohort A were consistent with that reported in the CT18 study (ORR: 50%; median PFS: 7 months) in advanced NSCLC patients received toripalimab combined with chemotherapy after resistance to prior EGFR TKIs (7). Nevertheless, 75% of patients enrolled in our study had received third or later lines of therapy, including four patients with T790M positive and resistance to third-generation TKIs. In the cohort A, 6/8 patients received received ICI plus chemotherapy and anti-angiogenesis therapy, of which 4 cases (66.7%) achieved PR. IMpower150 (8) included patients with EGFR mutation treated with ACP (atezolizumab + carboplatin + paclitaxel) or BCP (carboplatin + paclitaxel + bevacizumab) or ABCP (atezolizumab + carboplatin + paclitaxel +bevacizumab). Our ORR appeared to be numerically higher than those reported in these three subgroups (16% *vs.* 18% *vs.* 24%, respectively). The numerically lower PFS in cohort A, compared with that of ABCP group in IMpower150 study, may be explained in part by the inclusion of 2 patients who received ICI-chemotherapy without anti-angiogenic drugs.

The adverse-event profile observed in our study was as expected on the basis of the known events, and no new safety signals were found. The present results reveal that the proportion of grade ≥3 AEs (25%) in cohort A was numerically lower than those reported in the CT18 (55%) (7) and IMpower150 (ACP *vs.* BCP *vs.* ABCP, 57% *vs.* 57% *vs.* 64%, respectively) studies (8). Additionally, there were no grade 3–5 immune-related AEs recorded in our study. The majority of AEs were resolved or improved and were manageable.

This study had some limitations. Firstly, it was a retrospective study performed in a real-world setting, with potential for bias. We did not perform subgroup analysis due to a small sample size. We are currently conducting relevant prospective clinical studies (NCT04316351, NCT04310943). Secondly, the present findings provide only a narrow time window with limited follow-up of some patients. As a result, we did not calculate OS, and were able to analyze ORR and PFS as measures of short-term efficacy. The follow-up results will provide a more comprehensive analysis.

In conclusion, we showed a clinically meaningful survival benefit and a lower rate of severe AEs in patients treated with ICIs plus “chemo-reform”. These clinically relevant data support the use of immunotherapy combined with single-drug chemotherapy may be a new treatment option for patients with advanced NSCLC after developing resistance to EGFR-TKIs. Prospective clinical studies are needed for further validation.

## Data Availability Statement

The raw data supporting the conclusions of this article will be made available by the authors, without undue reservation.

## Ethics Statement

The studies involving human participants were reviewed and approved by the Institutional Review Board of the First Affiliated Hospital of Guangzhou Medical University (Guangzhou, Guangdong, China). The patients/participants provided their written informed consent to participate in this study.

## Author Contributions

HD, XL, XX, and CZ designed the study. HD, YY, LW, and JW collected the patients’ data. HD, XL, ZX, ML, and YQ analyzed the data. HD, XL, XX, and CZ drafted and revised the manuscript. All authors contributed to the article and approved the submitted version.

## Funding

This study was supported by State Key Laboratory of Respiratory Disease-The open project (SKLRD-OP-202111), Beijing Xisike Clinical Oncology Research Foundation (Y-2019Genecast-076), and Beijing Bethune Charitable Foundation (BQE-TY-SSPC(5)-S-03).

## Conflict of Interest

The authors declare that the research was conducted in the absence of any commercial or financial relationships that could be construed as a potential conflict of interest.

## Publisher’s Note

All claims expressed in this article are solely those of the authors and do not necessarily represent those of their affiliated organizations, or those of the publisher, the editors and the reviewers. Any product that may be evaluated in this article, or claim that may be made by its manufacturer, is not guaranteed or endorsed by the publisher.
