# Supplementary material for: Immune Checkpoint Inhibitors Plus Single-Agent Chemotherapy for Advanced Non-Small-Cell Lung Cancer After Resistance to EGFR-TKI
Source: Front Oncol. 2021 Sep 20;11:700023. doi: 10.3389/fonc.2021.700023 (PMC8488293; doi:10.3389/fonc.2021.700023)
Supplement: Supplementary file 1 [file DataSheet_1.pdf]

The next generation sequencing data of the patients after EGFR-TKIs resistance						
Patient ID	Specimen type	Gene	Exon_Rank	Description	AF	Mutation_Type
A-3	FFP	PTEN	2	c.80-1G>C	0.2553	splice_acceptor_
A-3	FFP	SMARCA4	17	p.E821G	0.3891	missense_varian
A-3	FFP	RNF43	9	p.E746fs	0.5014	frameshift_variant
A-3	FFP	FAT3	7	c.4611+8C>A	0.2597	intron_variant
A-3	FFP	NOTCH1	34	p.A2553=	0.4227	synonymous_variant
A-3	FFP	LRP1B	80	p.G4075=	0.0873	synonymous_variant
A-3	FFP	EGFR	20	p.C797S	0.1207	missense_varian
A-3	FFP	EGFR	23	p.G930E	0.1034	missense_varian
A-3	FFP	EGFR	21	p.L858R	0.197	missense_varian
A-3	FFP	TP53	8	p.T284fs	0.4691	frameshift_variant
A-3	FFP	EGFR	20	p.T790M	0.1291	missense_varian
A-3	FFP	EGFR	NA	cn_amp	3.33	cn_amp
A-3	FFP	RAC1	NA	cn_amp	3.94	cn_amp
A-4	PLA	EGFR	19	p.E746_A750delELREA	0.0776	del
A-4	PLA	EGFR	20	p.T790M	0.0688	snv
A-4	PLA	TP53	8	p.G262Vfs*83	0.0672	snv
A-5	PLA	STAG2	30	p.R1033*	0.0211	stop_gained
A-5	PLA	TP53	5	p.Q136*	0.0107	stop_gained
A-5	PLA	EGFR	21	p.L858R	0.0075	missense_varian
A-6	PLA	EGFR	19	p.P753_I759delinsA	0.03	delins
A-6	PLA	EGFR	20	p.T790M	0.011	snv
A-6	PLA	RBM10	15	p.E624Nfs*145	0.006	del
A-7	FFP	HGF	14	p.C519=	0.3074	synonymous_variant
A-7	FFP	PRKDC	33	p.V1399E	0.7425	missense_varian
A-7	FFP	TP53	8	p.C277Y	0.7754	missense_varian
A-7	FFP	PDGFRB	22	p.N1019=	0.2849	synonymous_variant
A-7	FFP	CARD11	17	c.2143-19C>T	0.2513	intron_variant
A-7	FFP	ASXL1	12	p.L1488V	0.3009	missense_varian
A-7	FFP	AXIN1	6	p.P494=	0.6296	synonymous_variant
A-7	FFP	FGFR2	14	p.I623L	0.6331	missense_varian
A-7	FFP	BCL2	3	p.H238Q	0.5699	missense_varian
A-7	FFP	PIK3CB	2	p.W60R	0.4326	missense_varian
A-7	FFP	FGFR4	3	p.R78L	0.2578	missense_varian
A-7	FFP	EGFR	20	p.C797G	0.1222	missense_varian
A-7	FFP	EGFR	20	p.T790M	0.2473	missense_varian
A-7	FFP	EGFR	19	p.E746_A750del	0.9124	disruptive_inframe_deletion

A-7	FFP	IKZF1	4	p.S88=	0.0601	synonymous_variant
A-7	FFP	ERBB3	NA	cn_amp	5.4	cn_amp
A-7	FFP	GLI1	NA	cn_amp	5	cn_amp
A-7	FFP	EGFR	NA	cn_amp	15	cn_amp
A-7	FFP	MDM2	NA	cn_amp	5.4	cn_amp
A-7	FFP	NFKBIA	NA	cn_amp	37.5	cn_amp
A-7	FFP	NKX2-1	NA	cn_amp	60.9	cn_amp
A-7	FFP	CDK4	NA	cn_amp	5.3	cn_amp
A-7	FFP	ARID2	NA	cn_amp	4.9	cn_amp
A-7	FFP	ZNF217	NA	cn_amp	4.6	cn_amp
A-7	FFP	KMT2D	NA	cn_amp	4.8	cn_amp
A-7	FFP	FOXA1	NA	cn_amp	44	cn_amp
A-7	FFP	IKZF1	NA	cn_amp	11.6	cn_amp
A-7	FFP	PARP1	intergenic_Intron12	intergenic(LOC101927851,TOMM20)-PARP1	0.363	gene_fusion
A-7	FFP	RAD50	Intron3_Intron2	C5orf67-RAD50	0.3532	gene_fusion
A-8	FFP	EGFR	19	p.E746_A750del	0.121	del
A-8	FFP	TP53	5	p.Y163C	0.383	snv
A-8	FFP	EGFR	NA	cn_amp	1.8	cn_amp
B-1	PLA	EGFR	21	p.L858R	0.7016	missense_varian
B-1	PLA	EGFR	NA	cn_amp	4.49	cn_amp
B-2	PLA	EGFR	19	p.E746_A750del	0.0528	disruptive_inframe_deletion
B-2	PLA	EGFR	21	p.T790M	0.0059	missense_varian
B-2	PLA	NTRK1	NA	cn_amp	0.0209	cn_amp
B-2	PLA	TP53	10	p.Arg337Leu	0.0238	missense_variant
B-3	PLA	TP53	6	p.I195fs	0.0059	frameshift_variant
B-3	PLA	EGFR	19	p.L747_A750delinsP	0.0081	disruptive_inframe_deletion
B-4	FLD	MET	18	p.L1195F	0.0345	missense_varian
B-4	FLD	BRCA2	15	p.V2503=	0.0398	synonymous_variant
B-4	FLD	EGFR	21	p.L858R	0.0326	missense_varian
B-4	FLD	FGF4	3	p.D153N	0.033	missense_varian
B-5	FFP	MSH6	1	p.R26W	0.04	snv
B-5	FFP	PIK3CA	10	p.E542K	0.524	snv
B-5	FFP	EGFR	19	p.E746_A750del	0.26	del
B-5	FFP	TP53	5	p.A159V	0.609	snv
B-5	FFP	RAD51D	3	p.S62*	0.011	snv
B-5	FFP	BRCA2	NA	del	0.7	del
B-5	FFP	RB1	NA	del	0.7	del
B-5	FFP	ERBB2	NA	snv	1.9	snv
B-5	FFP	CHEK2	NA	del	0.7	del
B-6	FFP	TBX15	8	p.E427K	0.239	snv
B-6	FFP	LRP1B	23	p.E1175D	0.105	snv

B-6	FFP	EGFR	19	p.L747_T751del	0.478	del
B-6	FFP	TP53	IVS7-EX8	.	0.471	del
B-6	FFP	MCL1	NA	cn_amp	2.6	cn_amp
B-6	FFP	RAC1	NA	cn_amp	2.6	cn_amp
B-6	FFP	EGFR	NA	cn_amp	2.3	cn_amp
B-6	FFP	MET	NA	cn_amp	1.9	cn_amp
B-6	FFP	MYC	NA	cn_amp	1.7	cn_amp
B-6	FFP	CCND1	NA	cn_amp	1.9	cn_amp
B-6	FFP	NKX2-1	NA	cn_amp	2.0	cn_amp
B-6	FFP	NTHL1	NA	cn_amp	2.3	cn_amp
B-6	FFP	FANCA	NA	cn_amp	2.3	cn_amp
B-6	FFP	SMAD4	NA	del	0.6	del
B-6	FFP	CCNE1	NA	cn_amp	6.6	cn_amp
B-6	FFP	RUNX1	NA	cn_amp	2.4	cn_amp
B-6	FFP	TMPRSS2	NA	cn_amp	2.2	cn_amp
B-6	FFP	U2AF1	NA	cn_amp	2.0	cn_amp
B-6	FFP	EIF1AX	NA	cn_amp	2.4	cn_amp
B-6	FFP	BCOR	NA	cn_amp	2.1	cn_amp
B-6	FFP	KDM6A	NA	cn_amp	1.9	cn_amp
B-6	FFP	ARAF	NA	cn_amp	2.2	cn_amp
B-6	FFP	STAG2	NA	cn_amp	2.0	cn_amp
B-7	PLA	APC	NA	p.Val1822Asp	1	missense_variant
B-7	PLA	ATP10B	NA	p.Cys217Arg	0.998	missense_variant
B-7	PLA	BRCA2	NA	p.Asn372His	0.473	missense_varian
B-7	PLA	BRCA2	NA	p.Val2466Ala	0.9995	missense_variant
B-7	PLA	CNOT4	NA	p.Val548Ile	0.4899	missense_varian
B-7	PLA	CYP2D6	NA	p.Pro34Ser	0.502	missense_varian
B-7	PLA	DPP10	NA	p.Val405Ile	0.9998	missense_varian
B-7	PLA	ERBB2	NA	p.Ile655Val	0.4776	missense_varian
B-7	PLA	GKN2	NA	p.Ser113Asn	0.9999	missense_variant
B-7	PLA	KRTAP5-5	NA	p.Arg17Leu	0.9732	missense_varian
B-7	PLA	KRTAP5-5	NA	p.Ser157Cys	0.9987	missense_variant
B-7	PLA	NOTCH2	NA	p.Cys19Trp	0.5235	missense_varian
B-7	PLA	NOTCH4	NA	p.Leu13_Leu16de	0.8422	nonframeshift_variant
B-7	PLA	OR2T4	NA	p.Ile253fs	0.9842	frameshift_variant
B-7	PLA	OR4A15	NA	p.Pro26Ser	1	missense_varian
B-7	PLA	OR4A15	NA	p.Gln256*	0.5265	Nonsense mutation
B-7	PLA	OR4C6	NA	p.Ile133Thr	0.9998	missense_varian
B-7	PLA	PDE4DIP	NA	p.Ala1066Thr	0.4672	missense_variant
B-7	PLA	POM121L12	NA	p.Gln64Glu	0.9997	missense_variant
B-7	PLA	PSG5	NA	p.Leu18Val	1	missense_varian
B-7	PLA	PTEN	NA	p.Leu171Val	0.4459	missense_varian

B-7	PLA	TNN	NA	p.Pro930Le	0.5028	missense_varian
B-7	PLA	TP53	NA	p.Pro72Arg	0.4906	missense_varian
B-7	PLA	TRIM58	NA	p.Thr374Met	0.4926	missense_varian
B-7	PLA	UGT1A1	NA	p.Gly71Arg	0.5044	missense_varian
B-8	PLA	EGFR	20	p.C797S	0.0014	missense_varian
B-8	PLA	EGFR	21	p.L858R	0.0037	missense_varian
B-8	PLA	EGFR	20	p.T790M	0.0038	missense_varian
B-8	PLA	POLE	NA	cn_amp	2.8	cn_amp
B-9	PLA	EGFR	19	p.E746_T751delinsA	0.0243	disruptive_inframe_deletion
B-9	PLA	EPHA5	3	p.T150I	0.0065	missense_varian
B-9	PLA	PTPRD	28	p.T980=	0.0071	synonymous_variant
B-9	PLA	EGFR	20	p.T790M	0.0124	missense_varian
B-9	PLA	TP53	7	p.C238F	0.0121	missense_varian
